# GIGA: a simple, efficient algorithm for gene tree inference in the genomic age

**DOI:** 10.1186/1471-2105-11-312

**Published:** 2010-06-09

**Authors:** Paul D Thomas

**Affiliations:** 1 Evolutionary Systems Biology Group, SRI International, Menlo Park, CA USA; 2 Division of Bioinformatics, Department of Preventive Medicine, University of Southern California, Los Angeles, CA USA

## Abstract

**Background:**

Phylogenetic relationships between genes are not only of theoretical interest: they enable us to learn about human genes through the experimental work on their relatives in numerous model organisms from bacteria to fruit flies and mice. Yet the most commonly used computational algorithms for reconstructing gene trees can be inaccurate for numerous reasons, both algorithmic and biological. Additional information beyond gene sequence data has been shown to improve the accuracy of reconstructions, though at great computational cost.

**Results:**

We describe a simple, fast algorithm for inferring gene phylogenies, which makes use of information that was not available prior to the genomic age: namely, a reliable species tree spanning much of the tree of life, and knowledge of the complete complement of genes in a species' genome. The algorithm, called GIGA, constructs trees agglomeratively from a distance matrix representation of sequences, using simple rules to incorporate this genomic age information. GIGA makes use of a novel conceptualization of gene trees as being composed of orthologous subtrees (containing only speciation events), which are joined by other evolutionary events such as gene duplication or horizontal gene transfer. An important innovation in GIGA is that, at *every *step in the agglomeration process, the tree is interpreted/reinterpreted in terms of the evolutionary events that created it. Remarkably, GIGA performs well even when using a very simple distance metric (pairwise sequence differences) and no distance averaging over clades during the tree construction process.

**Conclusions:**

GIGA is efficient, allowing phylogenetic reconstruction of very large gene families and determination of orthologs on a large scale. It is exceptionally robust to adding more gene sequences, opening up the possibility of creating stable identifiers for referring to not only extant genes, but also their common ancestors. We compared trees produced by GIGA to those in the TreeFam database, and they were very similar in general, with most differences likely due to poor alignment quality. However, some remaining differences are algorithmic, and can be explained by the fact that GIGA tends to put a larger emphasis on minimizing gene duplication and deletion events.

## Background

Phylogenetic inference algorithms have a very long history [1]. The earliest algorithms used information about macroscopic phenotypic "characters" to determine the evolutionary relationships between species. So it was natural that as soon as genetic (DNA) or genetically encoded (protein) sequences became available, these were treated as "molecular characters" that could be used, essentially in an identical manner to phenotypic characters, to elucidate species relationships. Out of this *character evolution paradigm *were developed techniques such as the maximum parsimony algorithm [2], and various approximate methods that aim toward parsimony such as neighbor-joining (NJ) [3], as well as methods that assume constant "molecular clock"-like behavior such as the unweighted pair group method with arithmetic mean (UPGMA) [4]. More recently, a different paradigm has developed specifically for molecular sequences. This *sequence evolution paradigm *is exemplified by maximum likelihood (ML) [5] and Bayesian [6] methods, which use an explicit model of how molecular sequences change over time. Different possible evolutionary histories ("alternative hypotheses") are distinguished by their relative likelihood under a particular model of sequence evolution. This paradigm has also led to the use of "corrected" distances calculated using a sequence evolution model, as an input into distance-based methods such as NJ and UPGMA. Many of the important recent developments in phylogenetic inference have involved constructing ever more realistic models of sequence evolution. The increased accuracy has a price, though, both in the computational power required and in the complexity of downstream analysis, such as interpreting the resulting inferences and comparing alternative hypotheses from different models or parameter sets.

In the genomic age, knowledge of a "representative genome" for many different species provides the opportunity to consider yet another paradigm, which we dub the *genome evolution paradigm*. Recently, several approaches have been developed that make use of genomic information in the construction of gene trees. One common method is species tree reconciliation [7,8], which takes a gene tree (typically estimated using NJ, a character evolution paradigm method) and then prunes and rearranges branches (typically those with weaker statistical support) to reduce the number of implied gene duplications and losses given a known species tree. The soft parsimony algorithm [9] extends tree reconciliation to minimize duplications and losses given an uncertain species tree (containing "soft" polytomies or multifurcating nodes). The SPIDIR algorithm [10] extends the sequence evolution paradigm by learning lineage-specific rate parameters for phylogenetic reconstruction over a large number of orthologous gene trees simultaneously. The SYNERGY algorithm [11] constructs a gene tree by using a known species tree to specify the sequence of iterative steps--bottom-up from leaves to root--of building and rooting NJ trees. In addition to sequence dissimilarity, the distance used in the NJ step includes an empirical term to capture synteny, or shared genomic context, which provides long-range (extending over multiple genes) genomic sequence evidence of common descent. Synteny has been used in a number of gene tree inference algorithms [12], and results from the inheritance of contiguous sequence regions that include more than one product-encoding gene. Existing algorithms within the genome evolution paradigm have shown that including this additional information generally improves gene tree inference, but they are algorithmically quite complex and computationally expensive. We set out to ask the question: given the constraints that can be derived from knowledge of whole genomes, *how simple *can we make a gene tree inference algorithm? What is a minimal set of principles underlying the evolution of gene families needed to reliably reconstruct gene histories?

Note that the inference of gene trees in the genome evolution paradigm builds upon either the character or sequence evolution paradigms--as described above, sequence data retain a primary role in all such algorithms proposed to date. The difference is that, in the genome evolution paradigm, we can make use of information from whole genomes *in addition to *that which can be derived from information inherent in each gene itself. In GIGA, we make use of two additional sources of information, which are applicable for even very distant relationships (unlike synteny, which is not observed, for example, between the most distant animal lineages). First, in the genomic era, we have more accurate knowledge of the "true" species tree (insofar as the tree model holds, see discussion below). Whole genome sequences have provided important information for resolving many species relationships that were difficult to determine from physical characters, or from sequences of individual genes. Second, the genome sequence provides nearly complete knowledge of the genes in the genome (for protein-coding genes at least, given the current state of gene prediction). This is critically important for distinguishing between alternative hypotheses for gene trees, and for locating gene duplication events relative to speciation events. However, it is also important to acknowledge the limitations of the genome evolution paradigm for gene trees. Despite much rhetoric to the contrary, these are still early days in the genomic age. Gene predictions are not of uniformly high quality [13,14], and any inference algorithm must take steps to minimize errors arising from low-quality predictions.

In summary, our approach is to infer phylogenetic trees of gene families using 1) a "known" species tree, 2) knowledge of all recognizable members of a given family in each genome, and 3) identification of potentially problematic gene predictions, together with 4) some knowledge derived from the molecular sequences. Our hypothesis was that the genomic constraints and detection of potentially problematic sequences might allow the use of an extremely rudimentary representation of the sequences themselves. If so, we could develop a simple algorithm that would be straightforward to interpret and to improve in the future. We call our algorithm GIGA (Gene tree Inference in the Genomic Age), and it differs from existing genome evolution paradigm methods in that it does not, at any point, construct a tree using existing character or sequence evolution methods such as NJ or ML. Like the very simple, efficient UPGMA method, it builds up a gene tree iteratively using a pairwise sequence distance matrix. However, in stark contrast to UPGMA, the final tree topology from GIGA does not simply reflect the order in which sequence pairs are joined during the algorithm. Rather, the algorithm uses simple rules based on knowledge of evolutionary processes, for inferring the tree topology from the order of pairwise operations. In the following section, we describe these rules and the theoretical and empirical considerations underlying them, in the context of a novel conception of gene trees as being composed of orthologous subtrees (OS's) joined together by founding copying events (FCEs) such as gene duplication and horizontal transfer. We then describe the GIGA algorithm in detail. Finally, we assess the algorithm's performance by comparing to a comprehensive set of more than 14,000 phylogenetic trees from the TreeFam database.

## Results and Discussion

### Rationale behind the GIGA algorithm

In the spirit of making our initial algorithm as simple as possible, we designed a "greedy" algorithm that constructs a tree guided by the sequence distance matrix but additionally applying rules that aim toward a parsimony-like criterion minimizing the number of gene duplication and deletion events [15]. The algorithm iteratively joins together subtrees of sequences, beginning with the two sequences that are closest according to the distance matrix. The topology of the joined subtree after each iteration is not simply an agglomeration of the constituent subtrees; rather, rules are used to "rearrange" the joined subtree at each iteration, in accordance with additional (genomic) knowledge. In essence, at each stage of the agglomeration process, GIGA interprets the tree in terms of the evolutionary events (speciation and gene duplication) that most likely generated that tree.

We found that, somewhat surprisingly, we needed only a very rudimentary description of sequence distances to build accurate tree topologies. Our initial implementation uses simply the relative pairwise sequence difference: (*number of different amino acids at homologous sites*)/(*total number of homologous sites*). Furthermore, unlike other distance-matrix-based methods, our algorithm does not update distances after each step, but uses the "raw" sequence distances throughout (in effect, the distance between two groups is the minimum distance over all inter-group sequence pairs). These additional simplifications are possible because the rules described below strongly constrain the inferred evolutionary history; the sequence distances are required only to represent very approximately any actual sequence divergence from a common ancestor.

### Orthologous subtrees and gene trees

We first describe a novel conception of gene trees, which will simplify the explanation of our rules for determining the tree topology from the pairwise sequence distance-determined order of operations. In this conception, a gene tree is composed of "orthologous subtrees", i.e., containing sequences related by speciation events. Each OS contains at most one gene from each organism, and every sequence in the subtree is orthologous to the others. Distinct orthologous subtrees are joined together to produce a gene tree, via events that involve the copying (or transfer) of genetic material to create a new locus within an ancestral genome. When the copying is from the same genome (e.g., tandem gene duplication or whole genome duplication), the joining event is a gene duplication event; when the copying is from another genome, the joining event is a horizontal transfer event. In our representation, when a copying event occurs, one copy of the gene "remains" in the same OS as its ancestors, while the other copy "founds" a second OS. Each OS, then, has a "founding copying event", though at the root of the tree this event is unresolved. Thus, each OS is defined by 1) a relationship to the OS that contains the other duplicated copy ("sibling"), and 2) a date of the FCE, relative to speciation events in the sibling OS's.

**Figure 1:**
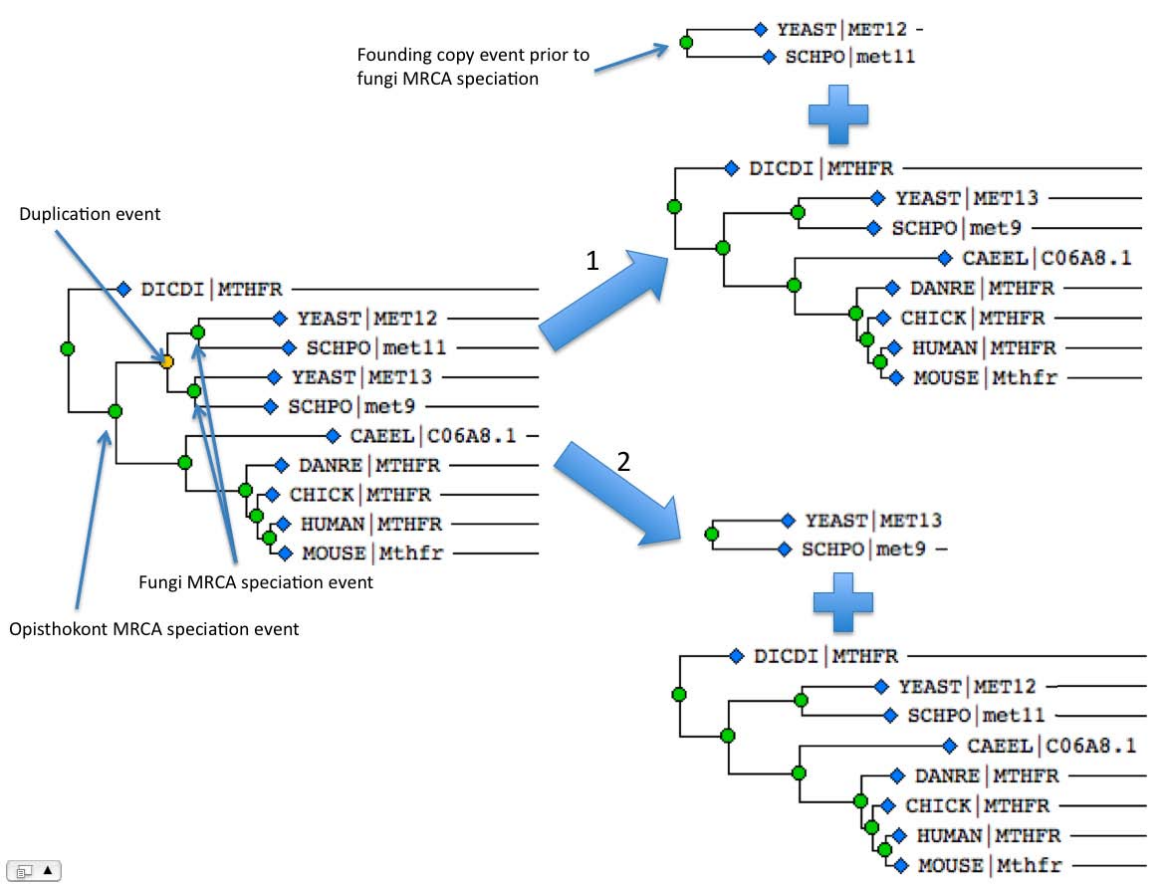
**Decomposing a tree with a duplication event into orthologous subtrees (OS's)**. The example shows part of the methylene tetrahydrofolate reductase (MTHFR in human) gene family. This tree can be decomposed into OS's in two different ways: 1) the fungal MET13/met9 group remains in the same OS as its ancestors, while the MET12/met11 group founds a new OS, and 2) the MET12/met11 group remains in the same OS as its ancestors, while the MET13/met9 group founds a new OS. In both cases, the two OS's are sibling groups, because they contain genes descending from the duplication event, and in both cases the FCE of the more recent OS (the one with only genes from fungi) can be dated relative to speciation events, between the opisthokont common ancestor and the fungal common ancestor in this example. Species are abbreviated with the 5-letter UniProt code: CAEEL (*C. elegans*, nematode worm), CHICK (*G. gallus*, chicken), DANRE (*D. rerio*, zebrafish), DICDI (*D. discoideum*, cellular slime mold), HUMAN (*H. sapiens*, human), MOUSE (*M. musculus*, mouse), SCHPO (*S. pombe*, fission yeast), YEAST (*S. cerevisiae*, Baker's yeast).

If there has been at least one copying event, there is more than one way to decompose a phylogenetic tree into OS's, depending on which copy is chosen to remain in its ancestral subtree, and which is chosen to found a new subtree. Each copying event can be decomposed in *n *possible ways, where *n *is the number of descendants of the copying event, so *n *= 2 for a bifurcating event. Thus, for a bifurcating tree with *N *copying events, there are *N*^2 ^possible decompositions into OS's. Figure 1 gives an example of a tree with one duplication event (orange circle) and the two possible ways in which it can be decomposed into OS's. Note that this example is designed merely to illustrate our conception that gene trees can be described in terms of OS's and the relationships between sibling OS's. The fact that the decomposition is not unique does not bear on our algorithm, as it *constructs gene trees from component OS's*, rather than decomposing a gene tree into constituent OS's.

### Rules for phylogenetic inference

With this representation we can describe rules for phylogenetic inference that can be applied during the distance-based iterative process. The first three rules describe how the species tree and genome content can be used to determine the topology of each OS (Rule 1), distinguish likely speciation from duplication events (Rule 2), and date FCEs relative to speciation events (Rule 3). The fourth rule enables initial OS's and FCEs to be revised at later steps in the process. The fifth rule attempts to minimize errors in tree reconstruction due to sequence fragments (usually due to partial gene predictions).

These rules treat only speciation and gene duplication events, i.e., vertical inheritance of genetic material (from parent to child). Less common, but still important particularly in prokaryotes, is "horizontal" gene transfer, in which DNA from a source other than a parent is integrated into the genome. In this case, the DNA being copied originates in another genome. However, it should be noted that vertical inheritance is generally treated as the null hypothesis even for bacterial genes, and horizontal inheritance is usually established by evidence that rules out vertical inheritance. We therefore focus in this paper on vertical inheritance, noting that there are already a number of methods for locating horizontal transfer events, such as incongruence with a vertical-only inheritance model [16] including comparison with ancestral sequence reconstructions [17], and extension of maximum parsimony to phylogenetic network representations [18].

#### Rule 1: if a subtree contains only speciation events, the topology is determined by the known species tree

When an ancestral species undergoes a *speciation event*, it is first separated into two reproductively isolated populations. Within the tree model of gene evolution, this event produces two copies of an ancestral gene, one in each species' genome, and these two copies then proceed to diverge from each other by well-known processes of population genetics, including mutation, random drift, and natural selection. *If only speciation events have occurred, and multiple speciation events do not occur within a relatively short period of time, the gene tree is expected to be congruent with the species tree*. Indeed, a major application of gene tree inference has been to infer species relationships. For genes that approximately obey "molecular clock-like" behavior such as ribosomal RNA genes [19] this remains a powerful tool. However, on a genome-wide scale, the inference of species trees based on single gene trees is notorious for giving different answers for different genes. While there are evolutionary scenarios, such as incomplete lineage sorting [20-22], by which a gene tree will be genuinely incongruent with the known species tree, recent studies have concluded that observed incongruence is much more often due to problems with sequence alignment algorithms, tree inference algorithms, and paucity of data when considering only relatively short regions of contiguous sequence, rather than to actual historical causes [10,23].

Particularly relevant to our approach, Rasmussen and Kellis [10] demonstrated that accounting for lineage-specific rate differences in a Bayesian evolutionary model dramatically increased the number of orthologous gene families among *Drosophila *species that matched the gene tree. Two of their important conclusions are that a single gene does not typically contain enough information to adequately resolve gene family relationships, and that lineage-specific differences in evolutionary rate (due to population dynamics) are a primary cause of incongruence between the species tree and gene tree. This is not to say that incomplete lineage sorting does not occur, or that a tree is always a good model for gene evolution. Incomplete lineage sorting may lead to cases where a gene tree genuinely disagrees with the known species tree, or agrees over some regions of a gene and not others (e.g., due to recombination); the reason for this disagreement is a breakdown of the gene tree model itself, which treats speciation and duplication as point events occurring to an ancestral genome, rather than as actual population-based events. Rather, these results suggest that for large-scale phylogenetic reconstruction, rate differences and inadequate information within a single gene may pose larger problems than incomplete lineage sorting. Therefore, in GIGA, we use the known species tree during the tree inference process to define a *species tree constraint *on the tree topology. Within the gene tree model, the problem of short speciation times can be addressed by simply allowing multifurcations in the underlying species tree. Finally, we note that even incorrect trees that assume the species tree topology is correct are useful as a null hypothesis for establishing that a more complicated evolutionary history has occurred.

#### Rule 2: a duplication event should be inferred only when there is *genomic proof *that a duplication occurred, *viz*. when, during the iterative process, a given subtree contains more than one gene from the same species (within-species paralogs)

**Figure 2:**
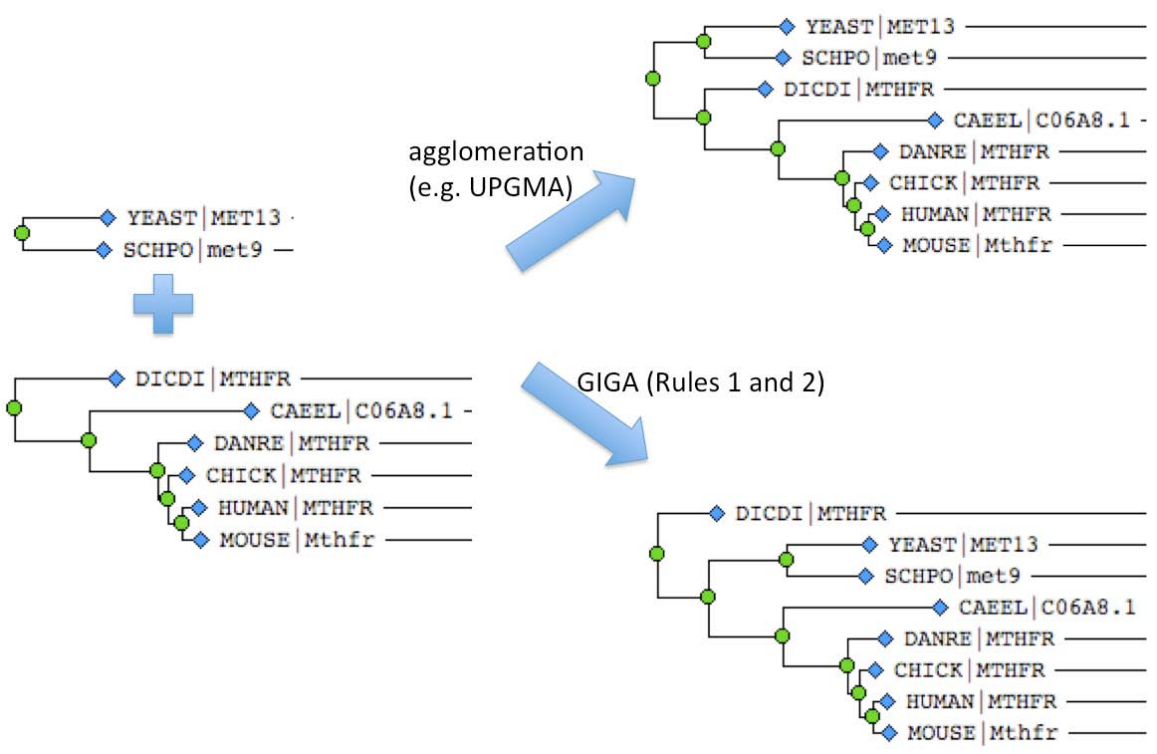
**GIGA rules for speciation events**. Note that GIGA Rules 1 and 2 result in a different tree topology than standard agglomerative methods such as UPGMA. Because GIGA uses knowledge of the species tree, it postulates that the yeast MET13/met9 group is actually orthologous to the MTHFR genes from other organisms, but was not merged prior to the sequence from *D. discoidieum *(DICDI) due to accelerated evolutionary rate in the fungal lineage.

As discussed under Rule 1, we do not expect phylogenetic inference to be accurate when using information from typical gene-length sequences, and we cannot then expect the agglomerative process, in general, to construct an orthologous tree in the order of the known species relationships. Therefore, when two OS's are joined at a given stage of the algorithm, if together they contain only a single gene from each genome, we merge the OS's into a single OS (Figure 2). Our simple rule assumes that the genes are in fact orthologous, but the sequence data was not adequate for recognizing this relationship. However, if the OS's together contain more than one sequence from any genome, our algorithm retains the two separate OS's, which are then joined by a gene duplication event (Figure 2B).

This procedure is similar to the "witness of non-orthology" criterion used by Dessimoz et al. [24] to identify paralogous relationships in the Clusters of Orthologous Groups (COGs) database [25]: within-species paralogs (z, z') can establish paralogy between pairs of genes (x, y) in other species if the distance *d*(x, y)>*d*(x, z) and *d*(x, y)>*d*(x, z'). This criterion is justified by the improbability of overall convergent evolution in molecular sequences. Most molecular sequence evolution is selectively neutral [26], and therefore similarity between molecular sequences is due more to common ancestry than to common selective selective pressures driving sequence convergence. If x is more similar to z, and y is more similar to z', than x is to y, this is almost certainly due to the fact that x and z have a more recent common ancestor than x and y; and y and z' have a more recent common ancestor than x and y. In other words, the two paralogous genes in genome Z, together with pairwise sequence distances, allow us to recognize that x and y are also paralogous. Thus, this is a genome age criterion, and can be used only if we know which genome each of the sequences came from, and if we can assume that our list of genes from each genome is nonredundant. Of course, relatively rare events, such as gene conversion or complementary deletions of paralogs in different genomes, can invalidate this assumption, and further rules could be developed to identify such cases.

#### Rule 3: if two subtrees of orthologous genes are related by a founding copy event, tentatively date the FCE using the gene content of the two groups and the known species tree

Given a known species tree, *if there have been gene duplication events, our task is to determine where each duplication event occurred relative to the speciation events*, i.e., which ancestral gene was copied, and when it was copied. In a character or sequence evolution paradigm, we must infer the location of duplications from sequence divergence. However, if evolutionary rates differ significantly for different lineages, parsimony and related approaches suffer from artifacts such as "long branch attraction," while likelihood methods typically make assumptions about the evolutionary model such as a constant relative substitution rate at each site. Yet evolutionary rate change is one of the prominent features of gene families, particularly after gene duplication [27]. After a duplication event, at least one copy is free to diverge under relaxed selective constraints and/or positive selection for a new or modified function; in a gene tree model this commonly manifests as branch- or lineage-specific accelerated evolutionary rate that differs among sites. We propose below that gene duplication events can be located using, in addition to sequence data, gene presence or absence over a particular set of genomes (genome content, used to construct "phylogenetic profiles" [28]) and knowledge of the species tree.

**Figure 3:**
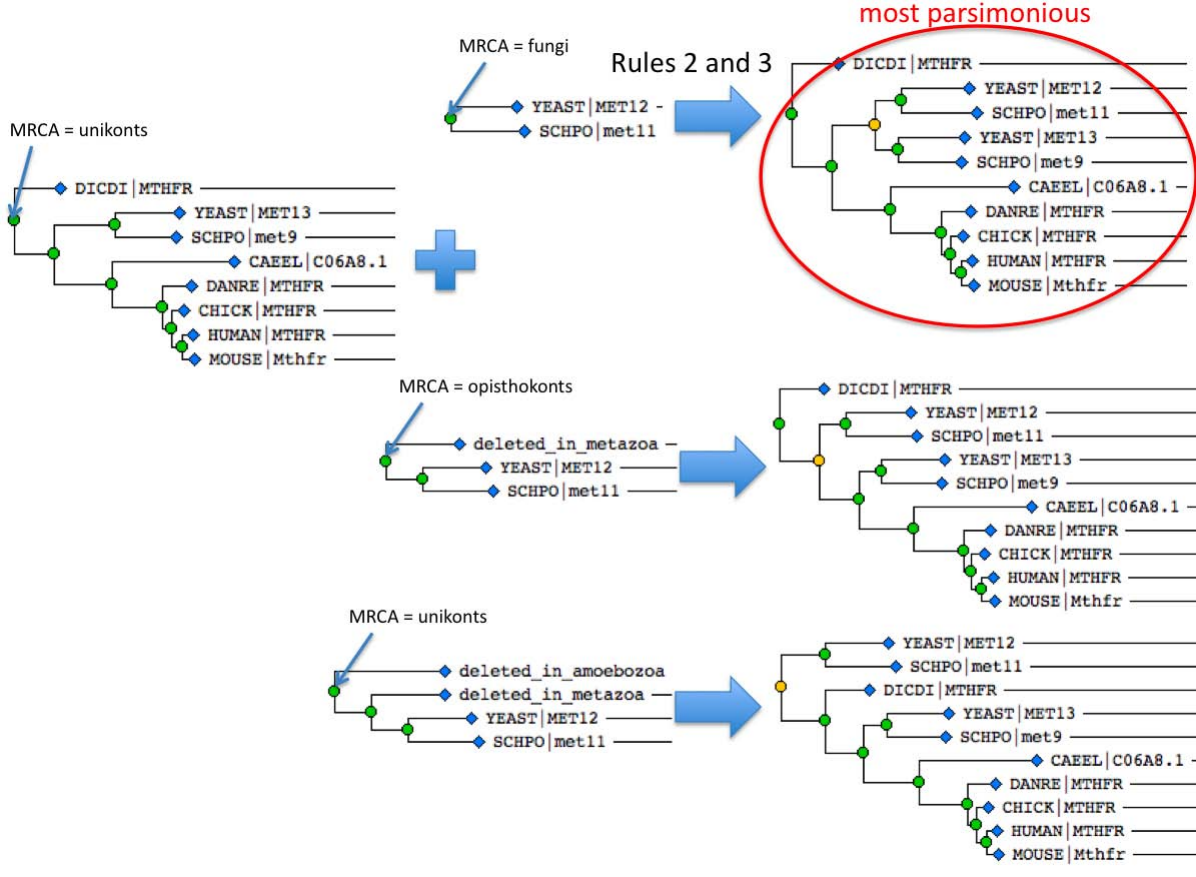
**GIGA rules for duplication events**. GIGA infers that a duplication must have occurred using Rule 2, as the two OS's being joined contain two genes from both Baker's yeast (YEAST) and fission yeast (SCHPO). It then places the duplication just prior to the most recent MRCA speciation event (fungi, in this case), which is the most parsimonious solution with respect to gene deletion events (Rule 3). Note that many other solutions are possible (two examples are shown below the most parsimonious case), but they require an increasing number of independent gene deletion events.

At the point at which OS's are inferred to be related by gene duplication (Rule 2), we have inferred the two descendant sibling lineages of the duplication. This specifies *which sequences *arose from a duplication. Because of two constraints--namely, the species tree, and the improbability of convergent sequence evolution--we can also make an initial hypothesis as to *when *the gene duplication may have occurred. Because, as described above, overall convergent sequence evolution is extremely rare, at the point in the iterative process where two OS's (inferred from shorter sequence distances) are joined by a duplication event, this duplication event very likely occurred prior to the most recent MRCA speciation event in either OS. The most recent common ancestor (MRCA) speciation event can be determined for each OS, from the species tree and the list of species with a gene in the OS. Thus, the OS must be older than its MRCA speciation event. We can therefore tentatively assign an FCE to the OS with the more recent MRCA speciation event (Figure 3, top). If both OS's have the same MRCA speciation event (as would be expected, assuming approximately molecular clock-like behavior and no gene loss), both OS's can be tentatively assigned an FCE. Note that this method of locating the FCE is reliable only if we know the full complement of genes in that family, for all the genomes under consideration; otherwise, the inferred founding ancestor for the subtree could depend on missing data rather than *established absence *of a gene.

Note also that gene loss may have occurred within an OS, which can cause the MRCA speciation event of all the *extant *sequences in the OS to be an underestimate of the age of the founding event (Figure 3, middle and bottom). However, we note that these alternative evolutionary histories become increasingly less likely as we go further back in time, as they invoke an increasing number of independent gene loss events. Thus, in the absence of additional information, the most parsimonious explanation of the data with respect to implied gene deletion events [15] is to connect each pair of related OS's according to the most recent FCE. Of course, additional information (such as synteny) could be used to revise this estimate, but in our simple algorithm we report only the most parsimonious reconstruction. This criterion implicitly considers the genomic presence/absence of genes to be a more reliable data source than the molecular sequence evolution rate, as estimated from character or sequence evolution methods. Finally, we note that multiple OS's may have the same MRCA speciation event and relate to the same sibling OS. In this case, multiple duplications have occurred between the same speciation events, and in this first implementation of GIGA we allow these to remain unresolved multifurcations.

#### Rule 4: if the founding copy event of an orthologous subtree has already been dated, allow this date to be revised based on additional evidence

Gene loss near the FCE is not the only reason that the initial MRCA speciation event may be an underestimate of the actual age of the FCE. Accelerated evolutionary rates near the FCE of an OS will also result in an underestimate. However, unlike gene loss (where we would need additional information such as synteny to recognize these events), we should be able to recognize most cases of accelerated rates in later iterations of the algorithm, and revise the FCE accordingly. This revision is necessary because we initially date an FCE when paralogous sequences are joined (Rules 2 and 3, Figure 3). If a lineage near the true FCE is accelerated in evolutionary rate, sequences in this lineage may have diverged more from their orthologs in other species than those orthologs have diverged from genuine paralogs. As a result, the sequence distances between paralogs will be smaller than those between some ortholog pairs, and the orthologs will be joined at a later iteration than the paralogs.

We can recognize possible cases of accelerated rate near the FCE in the following way. Even if there has been an accelerated evolutionary rate, there is likely to be some signal of common ancestry that can identify the diverged sequences as members of the correct orthologous subtree. We therefore ask whether these diverged sequences are *significantly *closer to this potentially orthologous subtree than to any other subtree. Consider a stage in the algorithm where the closest remaining pairwise distance asserts that OS1 (with a previously established FCE) should be merged with OS2 (containing potentially diverged orthologs) into a new OS. If OS2 contains genuine orthologs, these sequences will most likely retain sequence similarity evidence of this orthology. Recall that the FCE of OS1 was established due to a closer distance (earlier iteration) to a sibling OS containing at least one paralog of a sequence in OS1. Thus, if OS2 is significantly more similar to OS1 than to the sibling of OS1 (the closest paralogous group), this would be good evidence that it is actually orthologous to sequences in OS1. Because we calculate distance as the number of differences per site, we can simply use the Jukes-Cantor formula to estimate the standard deviation in this distance [29]. Depending on the alternative hypothesis to the proposed merge of OS1 and OS2, we take either one or three standard deviations to be significant, and if this criterion is met, the merge proceeds and the FCE is revised accordingly. If the alternative hypothesis is that OS2 is instead orthologous to the sibling of OS1 (which, like a merge with OS1 itself, also implies no gene duplications), we require the distance to be closer by at least *three *standard deviations. If the alternative hypothesis would require a gene duplication, either of OS1 or its sibling (i.e., OS2 is paralogous to the sibling of OS1), then we require less stringent evidence, namely, that the distance be at least *one *standard deviation closer.

#### Rule 5: if a sequence appears to be a fragment, leave it aside until the tree topology of all non-fragments has been determined

Obviously, it is of value to determine the evolutionary histories of as many genes as possible. However, it is well known that a nontrivial fraction of predicted genes in current genomes are partial predictions, which can cause problems for phylogenetic inference. Sequence fragments cannot be treated the same way as full-length sequences--e.g., for calculating pairwise distances or within a sequence evolution model--because different regions of a gene may be under dramatically different selective pressures, and will therefore evolve at very different rates; consequently distances estimated from part of the sequence may not accurately reflect those of the whole gene. One way to solve this problem is by constructing a multiple sequence alignment, and then restricting analysis to only those sites that are common to all sequences. However, this reduces the amount of data available for evolutionary inference, which as discussed above is already inadequate even if the entire gene sequence can be used.

It is not trivial, *a priori*, to distinguish a sequence fragment from a genuine evolutionary event in which a region of sequence has been gained or lost. For an evolutionary event, of course, we expect congruence with the phylogenetic tree: once a region of sequence is lost or gained in a particular ancestral gene, this gain or loss will be inherited by its descendants. Because, at a given stage in our tree reconstruction process, we have a hypothesis for the evolutionary history, we can make use of this expected congruence to identify potential sequence fragments on-the-fly. In our simple algorithm, each OS is a hypothesis about a group of sequences that descends from a common ancestor by speciation events, and we can expect to a good approximation that these sequences should have inherited most of the sequence sites present in the ancestor.

Thus, we implemented the following simple on-the-fly test for potential fragments. At a particular step in the iterative process, we are considering a possible merge between two OS's to form a new OS, based on a distance between two sequences. We want to avoid a merge if one (or both) of the sequences driving it is a sequence fragment, since in that case the merge would be based on unreliable data. We first approximate the sites likely to be present in the ancestral sequence as those columns of the multiple sequence alignment for which more than 50% of the sequences in the merged OS align an amino acid. We then test each of the two sequences driving the merge to identify each as a potential fragment. First, if the sequence is already part of an OS with at least three other sequences, it is not considered a fragment, since it passed our fragment test during previous steps, demonstrating that there are at least three other (presumably independently predicted, to some degree) orthologous sequences with similar structure. (The choice of three other independent observations is somewhat arbitrary, and of course depends on the number of species under consideration in a tree; in our tests below we considered as many as 50 species total, and varying this empirical parameter somewhat did not, in general, have an effect on the resulting trees.) If there are less than three other sequences in its OS, we gather all sequences from the potentially new, merged OS. If a sequence does not align more than 50% of the expected sites, it is identified as a potential fragment; the merge is not made; and the sequence is removed from the list of sequences to be used during the remainder of the iterative process. This prevents the fragment from determining the tree topology at any stage of the algorithm. However, we found that we could often correctly place sequence fragments during a second iterative process after a tree has been initially reconstructed for all non-fragment sequences. In this second process, each previously removed fragment is joined into the existing tree according to its shortest distance to a non-fragment sequence.

### The GIGA algorithm

The steps of the algorithm are as follows:

1. Preprocessing and setup

1.1 Decide on the genomes to be included; construct the "known" species tree for these genomes

1.2 For each protein family:

1.2.1 Assemble a "complete" set of genes for the given family.

1.2.2 Create a multiple sequence alignment of the genes in the family.

1.2.3 Select homologous sites in the alignment for sequence comparisons. We "trim" the alignment by removing a site if more than 15% of the weighted sequences are gapped at that site. Sequences are weighted using the procedure of Karplus et al. [30].

1.2.4 Represent sequence divergence at homologous sites. In the spirit of first trying the simplest model, we calculate the distance between each pair of sequences as simply the fraction of sequence differences at selected homologous sites.

2. For each protein family, infer the gene tree topology by iteratively defining orthologous groups, and how those groups are related via gene duplication events. Initialization: each sequence begins in its own OS.

**Figure 4:**
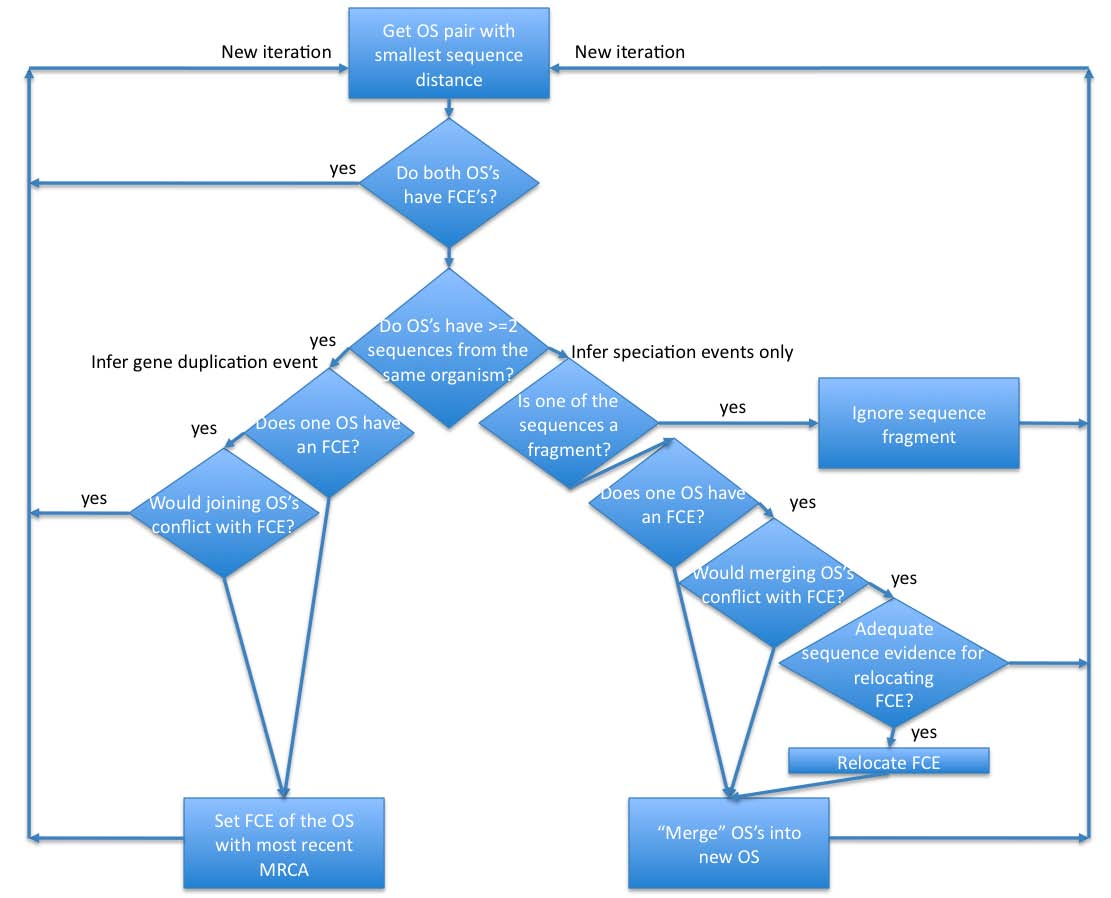
**Core of the simple GIGA algorithm**. OS = orthologous subtree, a portion of the gene tree containing only speciation events; FCE = founding copying event, the event (located relative to speciation events) that founded a given OS; in this first implementation of GIGA all FCEs are duplication events. The algorithm begins with each sequence in its own, separate OS. Each iteration operates on the currently closest pair of OS's. At each iteration, either 1) the two OS's are merged into a single OS (right side), or 2) one (or both) OS's are assigned FCEs. The tree is complete when all OS's have been assigned FCEs.

2.1: Consider the closest pair of sequences that has not been treated in previous steps, and do one of the following operations with the two OS's containing them (Figure 4):

2.1.1 Join the two OS's by a duplication event, and locate the event relative to the speciation events in each OS (Rule 3). If the two OS's, taken together, have two genes from a single organism, then they will be joined by a duplication event (Rule 2) if either of the following conditions also holds:

2.1.1.1: The founding duplication event has not been previously located for either OS.

2.1.1.2: The founding duplication event has been previously located for only OS1 and not OS2, and joining the two OS's will not conflict with this location. In other words, the phylogenetic span of OS2 must be less than or equal to that of OS1. This constraint means that joining the two OS's would not require us to revise an earlier hypothesis about when a duplication event occurred.

The founding duplication event is initially estimated so as to minimize the number of implied deletions, i.e. the FDE of the OS with the more recent MRCA is set to be immediately prior to that MRCA (Rule 3, Figure 3).

2.1.2: Merge the two separate OS's into a single OS (Rule 1).

2.1.2.1 Allow the merge only if the sequences are not likely fragments (Rule 5). If neither sequence is a fragment then the two OS's are merged if one of the following conditions holds:

2.1.2.2 The founding duplication event has not been located for either OS.

2.1.2.3 The founding duplication event has been located for only OS1 and not OS2, and merging the two OS's will not conflict with this location. In other words, the MRCA speciation event of the merged OS is the same as for OS1. This constraint means that merging the two OS's would not require us to revise an earlier hypothesis about when a gene duplication event occurred.

2.1.2.4 The founding duplication event has been located for only OS1 and not OS2, and merging the two OS's will conflict with this location, but there is adequate sequence evidence to support the revised location of the duplication event (Rule 4). We first calculate the standard deviation of the distance between OS2 and OS1 (dist1 and std_dev1) and that of the distance between OS2 and the sibling of OS1 (dist2 and std_dev2). If OS2 and the sibling of OS1 have no species overlap and might be orthologous, we require that

dist1-dist2>1.5(std_dev1+std_dev2)

Otherwise, we require a less stringent criterion that

dist1-dist2>0.5(std_dev1+std_dev2)

2.2 Attempt to add fragments back into the tree. Allow each fragment one attempted merge or join event, based on the shortest distance between the fragment and any non-fragment.

3. Infer tree branch lengths

We recommend taking the tree topology generated by GIGA and estimating branch lengths and ancestral sequences using an ML-based procedure, e.g. PAML [31]. However, in the spirit of the simple algorithm, we compute by default an approximate reconstruction of each ancestral sequence (a local, parsimony-like algorithm that reconstructs each node using only its descendants and closest outgroup), and then compute branch lengths as the sequence difference between adjacent nodes in the tree, including the Jukes-Cantor correction [29].

3.1 Infer ancestral sequences at each node. We do this in a simple manner, by recursion beginning at the leaf nodes (only extant sequences, the leaves, are known). For each non-leaf node, we consider the descendant nodes and its closest outgroup node. If the sequence of the closest outgroup node has not yet been determined, use its descendants to define the outgroup. If over half of the descendant nodes align the same amino acid at a given site, it is inferred to be the most likely ancestral amino acid. If the descendants disagree, and the outgroup agrees with one of them, the outgroup amino acid is inferred to be the most likely ancestral amino acid. Otherwise, the ancestral amino acid is considered to be unknown ('X').

3.2 Calculate branch lengths from node sequences. We use a simple measure, the fraction of sequence differences between a parent node and a child node. The Jukes-Cantor correction is applied to this value. However, in one respect we want to be very careful, and calculate distances only over a selected subset of sites. Following a duplication event, it is often the case that one of the duplicates continues to conserve the ancestral function more closely, while the other diverges more rapidly. We can identify the "least diverged" ortholog by tracing the shorter branch. Because of rate heterogeneity among sites, the relative branch lengths are reliable only if they are calculated over the same sites. Therefore in our algorithm, for branches following a duplication event, lengths are calculated using only those sites that are aligned in all the descendant nodes.

### Implementation

The GIGA algorithm has been implemented in the C programming language, and the code is available at ftp://ftp.pantherdb.org/.

### Testing the GIGA algorithm

Three properties of a phylogenetic inference algorithm are important to assess: *speed, accuracy *and *robustness*. Speed (compute time required to build each tree) should be assessed over a range of conditions to also determine how well a method scales with increasing number of sequences or alignment length. Robustness describes the sensitivity of the tree topology to perturbations such as adding/removing sequences, "resampled" character states (for calculating "bootstrap" values), or different parameter settings. Accuracy describes how well the inferred tree matches the actual history of evolutionary events. Of course, for actual gene families, we cannot go back in time and follow the "true" sequence of events, to know it for certain. In practice, accuracy can potentially be assessed in two ways: comparison against sequence data generated by "forward" evolutionary simulation for a known tree topology, or comparison with "gold standard" phylogenetic reconstructions. Simulated data sets are widely used, but their relevance for assessing gene tree inference algorithms is not established; the ability of an algorithm to correctly infer the underlying tree may be more dependent on how well it matches the particular simulation algorithm than how well it will work on actual gene data. On the other hand, there are as yet no "gold-standard" sets of diverse gene phylogeny reconstructions based on actual data. Several groups have used congruence with the "known" species tree as a gold-standard measure [10,32], but because GIGA uses such congruence as a constraint, this is not an appropriate test (though it does support the use of species tree congruence as a constraint).

**Figure 5:**
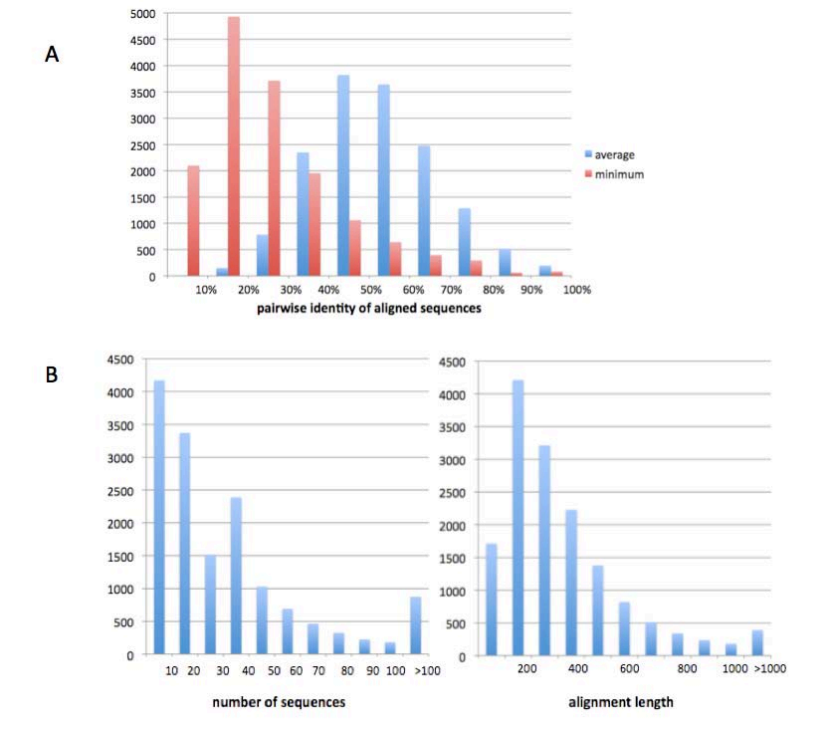
**Characteristics of the TreeFam families used in this study (14,331 families with at least 4 sequences)**. (A) Distribution of average and minimum pairwise identity of families, (B) Distributions of number of sequences and protein alignment length.

We suggest that the TreeFam resource [33] can be used to provide benchmarks for speed, accuracy and at least one type of robustness, namely the effect of adding more sequences (taxa) of potentially lower quality. TreeFam contains a large, diverse set of protein families. Moreover, most families display substantial sequence divergence, indicating that they do not represent trivial cases for evolutionary reconstruction. Figure 5 shows the distribution of average and minimum pairwise sequence identity across TreeFam protein alignments (considering only the positions that are aligned for most sequences, as described in the GIGA algorithm description 1.2.3 above). Average pairwise identity is approximately normal, with a mean and standard deviation of 52%+-15%, while the minimum pairwise identity mode is less than 20%, and nearly all families (>90%) have a minimum pairwise identity less than 50%. Figure 5B shows the distributions of the number of sequences in the TreeFam families (>4 sequences), and the lengths of the protein alignments.

Accuracy cannot be assessed directly (because the true evolutionary history is unknown), but consistency with TreeFam trees can be easily assessed. The quality of TreeFam trees on average has been established using a number of different metrics [34], so we can reasonably expect that an accurate method should produce trees similar to TreeFam trees in general. Because there is only one possible topology for a tree of two sequences, and only three possible topologies for a bifurcating tree of three sequences, we confined our analyses to 14,331 TreeFam families (release 6.1) comprising four or more sequences. Finally, robustness of an algorithm to perturbations such as adding sequences, and variations in sequence quality, can be assessed by comparing trees constructed from the two different TreeFam alignments, the "clean" and "full" alignments, which differ only in that the full alignments contain additional sequences from partially sequenced genomes. A perfectly robust method will infer identical trees for the sequences in the "clean" alignment, regardless of whether or not the additional "full" sequences are also included during the tree building process.

#### Algorithm speed

**Figure 6:**
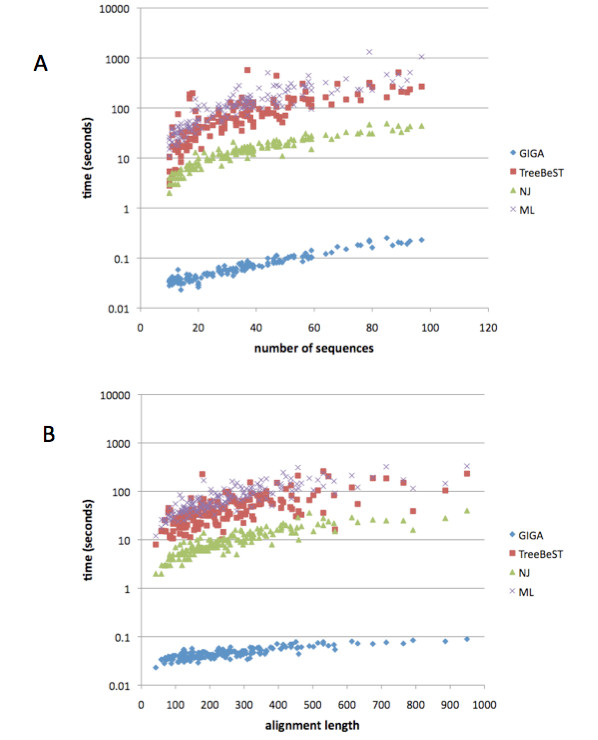
**CPU time required for tree reconstruction, note the log scale**. GIGA is over 100 times faster than NJ and 1000 times faster than ML methods. (A) Dependence on number of sequences (alignment length is constant at 200-204). (B) Dependence on alignment length (number of sequences is constant at 20). The same alignments are used for each method.

In order to assess algorithm speed and scaling, we selected two sets of families: one with families of the same alignment length (to test the algorithms' dependence on the number of sequences), and one with families of the same number of sequences (to test the algorithms' dependence on the alignment length). In the current implementation, GIGA scales similarly to other, commonly used methods in terms of dependence on the number of sequences and alignment length (Figure 6), but is over 100 times as fast as neighbor joining (as implemented in PhyML), and over 1000 times as fast as the ML methods PhyML and TreeBeST.

#### Accuracy of GIGA trees: consistency with TreeBeST

**Figure 7:**
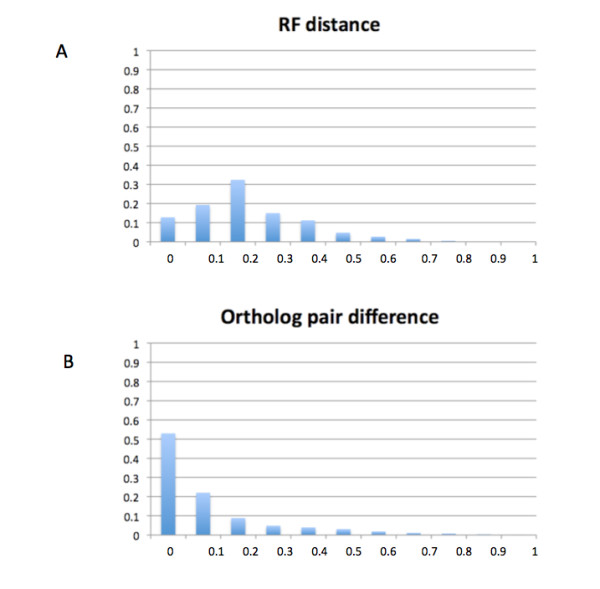
**Accuracy of GIGA trees: comparison with TreeFam clean trees for more than 14,000 TreeFam families**. A) normalized RF distance comparing tree topology; B) ortholog pair difference (see text) is substantially smaller than the RF distance, indicating that many of the topological differences between TreeBeST and GIGA trees are due to disagreements in speciation event order.

As a proxy for accuracy, we compared GIGA trees directly to TreeFam "clean" trees (the trees considered to be of highest quality in TreeFam). Figure 7A shows the Robinson-Foulds (RF) [35] distances (the most commonly used measurement of tree similarity) between TreeFam clean trees, and GIGA trees inferred using the same alignment. Overall, the trees produced with the two different algorithms are quite similar, with about 13% of the trees being identical and 64% very similar (RF distance < 0.2).

**Figure 8:**
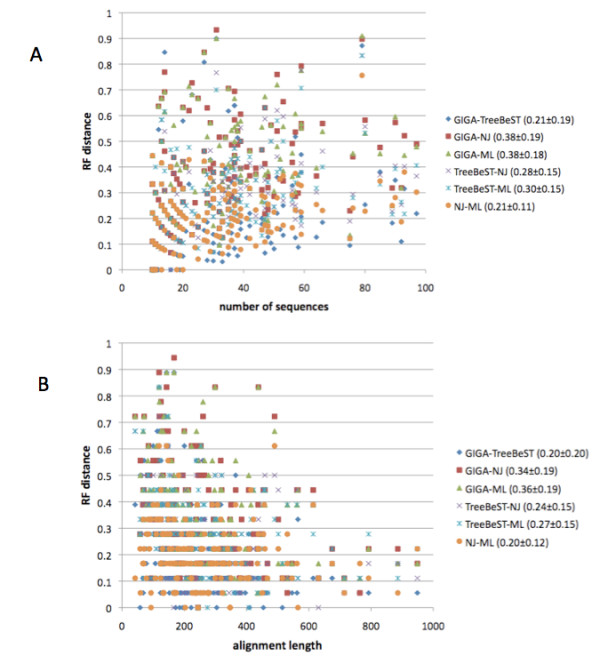
**Comparison of multiple different tree reconstruction methods**. The RF distance of each pair of trees is plotted versus (A) the number of sequences in the family and (B) the length of the alignment to show the dependence on these parameters. GIGA and TreeBeST (blue diamonds) generally yield more similar trees than any other pair of methods, except for NJ-ML, which is of comparable similarity. The RF distance mean and standard deviation for each pair of methods is in the figure legend in parentheses. The same subsets of TreeFam families was used as for Figure 6.

To further characterize the magnitude of these RF distances, we constructed both NJ and ML trees from the clean alignments using the PhyML program [36]. We then compared the trees from all four methods. While the RF distance distributions for all comparisons are affected by both the number of sequences and the alignment length, GIGA and TreeBeST trees are the most similar to each other in almost all cases, except for a few of the smaller trees (Figure 8). On average, GIGA and TreeBeST produce the most similar trees, along with NJ-ML (average RF = 0.21). The overall NJ-ML similarity is expected, since in PhyML the ML tree construction process begins with the NJ tree, so the comparable GIGA-TreeBeST similarity is striking.

For all comparisons, the RF distance correlates with the number of sequences when alignment length is constant (Figure 8A, R = 0.25 to 0.41 except GIGA-TreeBeST), though the GIGA-TreeBeST distance depends much less upon the family size (R = 0.16), presumably due to the species tree guidance. Thus, despite a slight absolute decrease in GIGA-TreeBeST similarity for larger families, this decrease is small relative to all other comparisons. Also for all comparisons, the RF score is negatively correlated with alignment length when the number of sequences is held fixed (Figure 8B, R = -0.25 to -0.37 except for NJ-ML), though this effect is much less pronounced for the NJ-ML comparison (R = -0.14) presumably because the NJ tree is used in the ML process. Importantly, all of the different methods tend to converge towards each other as the amount of substitution data increases. This is consistent with the conclusion of Rasmussen and Kellis [10] that shorter genes often lack sufficient information for accurate evolutionary reconstruction. Finally, because GIGA joins the closest sequence pair at each step, it is useful to compare it to the UPGMA method. On average, the UPGMA trees (estimated by PHYLIP [37], using corrected distances from PROTDIST using the JTT model [38]) are much less similar to TreeBeST trees (average RF = 0.30), and perhaps surprisingly, are even less similar to GIGA trees (average RF = 0.41) than to TreeBeST trees. Thus, on real protein family alignments, the tree construction rules in GIGA tend to predominate over the algorithmic ordering of joining operations, and these rules dramatically improve the match with TreeBeST trees.

Although the differences between GIGA and TreeBeST trees were not very substantial (no larger than those between PhyML and its NJ starting point, as discussed above), we explored these differences further. Because TreeFam trees use the species tree only as a "soft" constraint, we reasoned that some of the disagreement between the two algorithms was simply due to local rearrangements of speciation events, as opposed to more substantive differences in the location of gene duplication events. We can quantify this disagreement by comparing the sets of ortholog pairs that are inferred from the two trees. Because two genes are inferred to be orthologous if their most recent common ancestor in a gene tree is a speciation event, a local rearrangement involving only speciation events will have no effect on the inferred ortholog pairs. Differences involving duplication events, on the other hand, will affect the inferred ortholog pairs. We therefore calculated ortholog pairs for all the trees. We defined the ortholog pair difference as

1-(*number of pairs inferred in common from both trees*)/(*total number of pairs inferred from either tree*)

Figure 7B shows that for 53% of the TreeFam families, the inferred orthologs are in perfect agreement; in these cases the GIGA and TreeFam trees are either identical or display only minor rearrangements of speciation nodes relative to each other. For the vast majority of trees (84%), the ortholog difference is less than 0.2, suggesting generally good agreement on the inference of gene duplication events.

Because multiple sequence alignment quality is well known to influence phylogenetic reconstruction, we reasoned that some of the remaining discrepancy between TreeFam and GIGA trees might be due to poor alignment quality. We ran PredictedSP [39] on all TreeFam alignments to generate an alignment score (1 indicating perfect quality, with smaller numbers indicating lower quality). We found that families for which GIGA and TreeFam differed more substantially (ortholog pair difference > 0.2) had a strong tendency to have poor alignments. Over half (54%) of families with substantially different trees inferred by the two different algorithms (ortholog pair difference > 0.2) had a PredictedSP score of less than 0.85, while this was true of only 19% of the families with similar trees (ortholog pair difference < 0.2). This strongly suggests that poor alignment quality accounts for a substantial fraction of the discrepancies between TreeBeST and GIGA trees.

**Figure 9:**
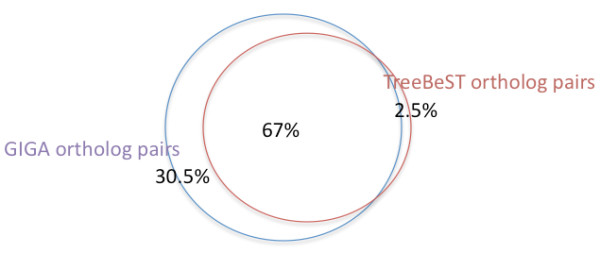
**Overlap between orthologs computed from GIGA and TreeBeST trees**. GIGA infers 96% of orthologs inferred by TreeBeST, but also finds many additional orthologs, due mainly to minimization of implied gene duplication and deletion events.

**Figure 10:**
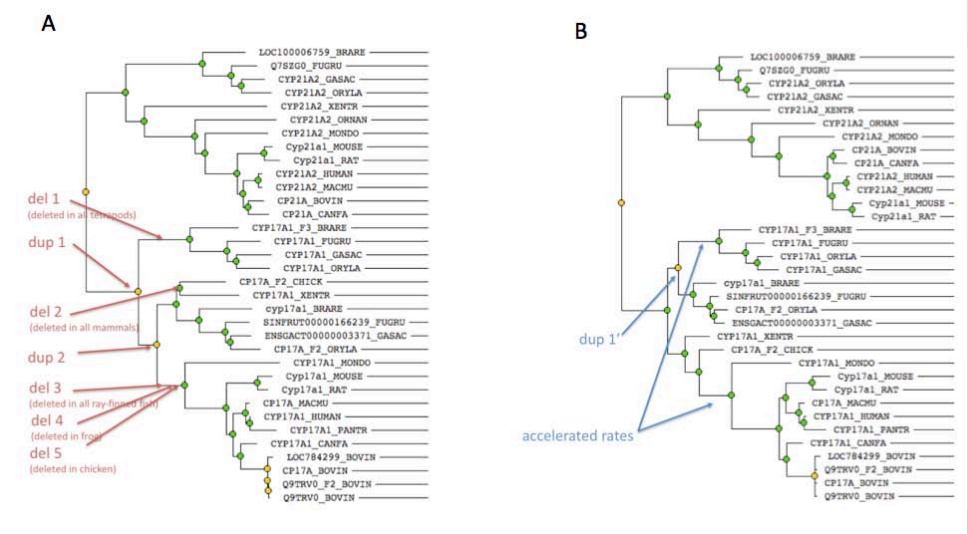
**Example of a tree with substantial disagreement in inferred duplication events, and corresponding orthologs, between TreeBeST (A) and GIGA (B), TreeFam family TF105095**. The sequence alignment is of high quality according to PredictedSP, so this disagreement is due to algorithm differences rather than a problematic alignment. The main differences are in the inference of gene duplication events (orange nodes) in the CYP17A1 lineage (other than the recent duplications in the bovine lineage). (A) TreeBeST infers two duplication events (dup 1 and dup 2), both prior to the ray-finned fish-tetrapod divergence, followed by at least five separate deletion events: one prior to the frog-amniote divergence (del 1), one prior to the chicken-mammal divergence (del 2), one prior to the fish radiation (del 3), one following the divergence of the frog lineage (del 4), and one following the divergence of the chicken lineage (del 5). Note that according to this tree, there are no orthologs of human CYP17A1 in chicken, frog, or fish. (B) GIGA infers one duplication event, before the fish radiation (dup 1') and no deletion events. Note that according to this tree, there is one ortholog of human CYP17A1 in frog, one in chicken, and two in each fish species. Note also that tree (B) infers two periods of accelerated (potentially adaptive) molecular evolutionary rates, which may account for why a molecular evolution model would favor a topology with longer divergence times such as in (A).

Poor alignment quality does not account for all the discrepancies. Despite the overall good agreement between predicted orthologs, there are systematic differences between the two tree inference methods. Over the entire set of more than 14,000 families we compared, 67% of all ortholog pairs inferred by *either *algorithm are in exact agreement. However, the disagreements are not randomly distributed between the two algorithms. GIGA infers a substantially larger number of ortholog pairs. Of all ortholog pairs inferred by TreeBeST, 96% are also inferred by GIGA, but of all ortholog pairs inferred by GIGA, only 69% are also inferred by TreeBeST (Figure 9). This difference can be largely explained by the fact that the GIGA algorithm locates duplication events using a genomic parsimony criterion, while TreeBeST also uses an ML sequence evolution model. GIGA will tend to locate duplication events as far as possible toward the leaves of the species tree, to minimize the number of implied deletion events. This, in turn, will enable a larger number of gene pairs to be traced to a common speciation event ancestor, i.e., a larger number of inferred orthologs. An example is shown in Figure 10.

#### Robustness of GIGA trees

**Figure 11:**
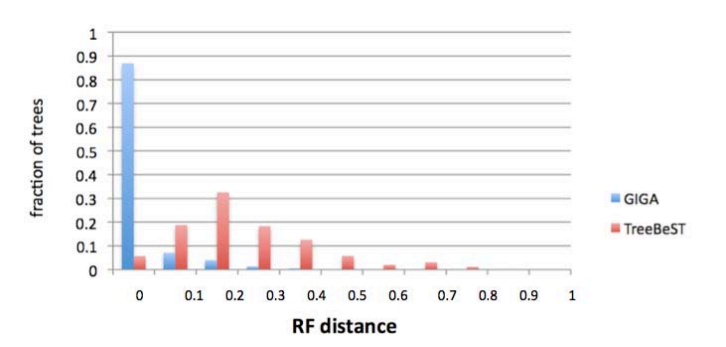
**Robustness of tree inference algorithms: histograms for GIGA and TreeBeST, for "clean" vs. "full" alignments for more than 14,000 TreeFam families**. Full alignments include additional sequences, but the alignment is the same as for the clean set. An RF distance of 0 indicates that the tree topology is unchanged by adding more sequences. Overall, GIGA is more robust than TreeBeST to the perturbation of adding sequences.

As a baseline, we first assessed the robustness of TreeBeST trees, by comparing the TreeFam "clean" and "full" trees. If the TreeBeST algorithm were perfectly robust to the addition of sequences, the topology of the TreeFam full tree would be identical (for the subset of sequences also in the clean tree) to the clean tree. To identify deviations from perfect robustness, we calculated the RF distance between the TreeBeST clean and full trees. Figure 11 (red bars) shows that TreeBeST is reasonably robust to the additional sequences. In relatively few cases are the TreeBeST trees for the clean and full alignments identical (5.7%) but most are very similar (57% have a distance less than 0.2; 88% have a distance less than 0.4). We note that the TreeBeST algorithm itself is somewhat different for full and clean alignments, as full trees are estimated using only protein sequences, while clean trees can use nucleotide as well as protein sequences.

To test the robustness of GIGA, we then constructed two separate GIGA trees for each TreeFam family, one from the "clean" protein alignment, and one from the "full" alignment. We then calculated the RF distance between the two GIGA trees to measure how much the additional "full" sequences changed the topology inferred for the "clean" sequences. We found that GIGA is considerably more robust than TreeBeST to the perturbation of adding sequences (Figure 11, blue bars), with over 85% of the trees being completely unchanged (RF distance of 0) and 98% changing in RF distance by less than 0.2. The robustness of GIGA is remarkable, and is due largely to the strong constraints provided by the rules described above.

## Conclusions

We described a simple algorithm, GIGA, for inferring the evolutionary events that have given rise to a particular gene family. We then demonstrated that this simple algorithm creates trees that are similar overall to those produced by the much more complex and computationally intensive TreeBeST algorithm [33]. We consider this to be evidence of the accuracy of GIGA, based a on published analysis of TreeBeST trees [34], though of course evolutionary reconstruction is an ongoing research activity. GIGA is over 1000 times faster than "fast" ML methods such as TreeBeST and PhyML, and over 100 times faster than neighbor joining. The GIGA algorithm can be simple precisely because it makes use of constraints on the evolutionary history that have only recently become available, with the advent of whole genome sequencing. The overall philosophy of the algorithm is that because of potentially dramatic departures from clocklike behavior in many gene families, even a fairly sophisticated treatment of molecular sequences is likely to be less trustworthy overall as a guide for constructing gene trees than is genome-derived information such as a known species tree, and gene content (presence or absence of particular genes). The algorithm does rely on sequences to reveal common ancestry (due to the improbability to convergent sequence evolution), even if the sequences alone may not reliably date that common ancestor. Of course, GIGA is not the ultimate implementation of this genome evolution paradigm--rather, it is only a simple first step.

The GIGA algorithm makes use of ideas that have been employed for some time. GIGA is similar to tree reconciliation [7,8] and soft parsimony [9], but rather than first estimating the entire tree and then reconciling it with the species tree, GIGA reconciles the tree at each step in the algorithm. Unlike soft parsimony, polytomies in the species tree or from rapidly repeated duplication remain unresolved, "simultaneous" events, in the current implementation of GIGA. Other algorithms have made use of a species tree to guide gene tree reconstruction, notably SYNERGY [11] and TreeBeST [33]. While SYNERGY uses the species tree to determine the order of iterative NJ tree building and rooting, GIGA builds up different "orthologous subtrees" (OS's) simultaneously and determines their relationships based on pairwise distances and genome content. While TreeBeST uses the species tree to count duplications and deletions, which are then treated in an ML framework with weighted probabilities relative to substitution events, GIGA minimizes the number of duplications and deletions consistent with the OS's, which is tantamount to giving these events a very large weight compared to substitutions.

One of the main advantages of the GIGA algorithm over other methods is its simplicity. This simplicity makes it particularly amenable to systematic improvement, as it is easy to identify the algorithmic reasons for the tree topology inferred by GIGA and to propose additional rules if necessary. In the future, one could develop rules to handle specific evolutionary events in addition to those addressed in this initial implementation, such as whole genome duplication, horizontal transfer, polytomies and even incomplete lineage sorting. Nevertheless, even with only the few rules presented here, the algorithm performs remarkably well for many applications.

As with nearly all computational methods, GIGA was designed to address certain applications of phylogenetic inference and is not appropriate for all applications. GIGA assumes that the "true" species tree is known (insofar as the tree model holds), and that we have a whole genome and "complete" knowledge of the genes in that genome. It is therefore applicable only to genomes that have been fully sequenced and annotated with respect to the genes in the families whose histories we wish to infer. It is obviously not amenable to analysis of gene sequences obtained by environmental sequencing (where the species of origin is not known), nor to inference of species phylogenies from gene sequences, nor to inference of incomplete lineage sorting (except as a null hypothesis). Nevertheless, the algorithm has many advantages for problems involving large-scale phylogenetic reconstruction, inference of orthologs, and inference of gene function by homology. It is very fast, enabling reconstruction of the phylogenies of large gene families. GIGA may even be appropriate as a starting point for refinement by "fast" maximum-likelihood methods. Finally, GIGA is remarkably robust to adding sequences. This property is particularly useful for phylogenomic databases, as it enables ancestral sequences to be referred to by a stable identifier over successive releases as new genomes are sequenced and new genes are annotated in existing genomes. In turn, the stable reference to ancestral sequences would enable the large-scale annotation of gene function by homology, explicitly tracing the evolution of gene function within a gene family. The Gene Ontology Reference Genomes Project is currently undertaking just such an effort, using the trees produced by the GIGA algorithm [40]. Trees produced by GIGA for 48 completed genomes are now available in the PANTHER version 7 database [41], which complements other existing phylogenomics resources that employ other tree reconstruction algorithms, such as TreeFam [33] (using a combined sequence/genomic event ML algorithm), PhylomeDB [42] (using NJ, ML and Bayesian algorithms) and GeneTrees [43] (using a Bayesian algorithm).

## List of abbreviations

FCE: founding copy event; GIGA: gene tree inference in the genomic age; ML: maximum likelihood; MRCA: most recent common ancestor; NJ: neighbor joining; OS: orthologous subtree; RF distance: Robinson-Foulds distance; UPGMA: unweighted pair group method with arithmetic mean.
